# Vascular Complications in Extracorporeal Membrane Oxygenation—A Narrative Review

**DOI:** 10.3390/jcm13175170

**Published:** 2024-08-31

**Authors:** Joseph P. Hart, Mark G. Davies

**Affiliations:** 1 Center for Quality, Effectiveness, and Outcomes in Cardiovascular Diseases, Houston, TX 77054, USA; mark.davies@ascension.org; 2 Division of Vascular and Endovascular Surgery, Medical College of Wisconsin, Milwaukee, WI 53226, USA; 3 Department of Vascular and Endovascular Surgery, Ascension Health, Waco, TX 76710, USA

**Keywords:** ECMO, pathophysiology, arterial complications, venous complications, mitigation, outcomes

## Abstract

The establishment of a peripheral ECMO circuit can lead to significant arterial and venous complications in 10–30% of patients. Vascular complications, particularly acute limb ischemia, are associated with worsening overall outcomes. Limb ischemia occurs significantly more frequently in the early stages of VA ECMO than in VV ECMO. Mechanisms of limb ischemia include arterial obstruction, cannulation injury, loss of pulsatile flow, thromboembolism, venous stasis from compressive obstruction with large venous cannulas, and systemic vasoconstriction due to shock and pharmacologic vasoconstriction. The care team may use several mitigation strategies to prevent limb ischemia. Arterial and venous complications can be mitigated by careful access site selection, minimizing cannula size, placement of distal perfusion and/or outflow catheter(s), and continuous NIRS monitoring. Rapid intervention, when ischemia or compartment syndrome occurs, can reduce limb loss but may not affect the mortality and morbidity of the ECMO patient in the long term due to their underlying conditions and the etiology of the ECMO need.

## 1. Introduction

Peripheral ECMO use is increasing to treat many acute cardiovascular events and for the management of respiratory failure to support cardiovascular circulation (VA-ECMO) and provide cardiopulmonary support (VV-ECMO) [1,2,3]. ECMO can exist as a standalone therapy, an adjunctive therapy, or a transition therapy to organ transplantation or mechanical device implantation [3,4,5,6,7]. Despite favorable results in many pathologies, ECMO can cause numerous complications that lead to significant morbidity and mortality [8,9,10,11]. Both central access and peripheral access ECMO are employed. The vascular complications associated with central ECMO are related to cannula dislodgement and mediastinal hematoma; they are well-recognized and are managed surgically. With respect to the vasculature in peripheral ECMO, ECMO can induce both arterial and venous injuries [12,13]. Once ECMO support commences, vascular and associated hematological complications may lead to acute limb ischemia, compartment syndrome, bleeding, embolism, and thrombosis. (Table 1) The management of vascular complications is associated with the need for additional resources to support the patient and the need for further procedures that may necessitate major amputation [14]. The occurrence of vascular complications is a marker of overall higher morbidity and mortality in those patients placed on ECMO. Literature reviews estimate that the incidence of vascular complications is around 10–30% of all adults placed on ECMO, with complications occurring far more commonly in VA-ECMO compared to VV-ECMO [13,15,16,17,18,19]. Vascular complications can be categorized as early or late. Early vascular complications are those that occur during ECMO, and late complications are those that occur after decannulation from ECMO [13].

## 2. Anatomy

The anatomy of the leg contributes to the vascular complications seen during an ECMO run. The limb receives its blood supply from the aorta through the iliac system to the common femoral artery. The internal iliac artery perfuses the pelvis and provides the first branch of the cruciate collateral system to the leg. Its ostium can be covered by a cannula. The normal size of the common iliac artery ranges from around 7 to 10 mm, and that of the external iliac artery, from 6 to 10 mm [20]. The normal diameter of the common femoral artery ranges from about 6 to 8 mm in adults. It is important to note that these diameters vary depending on age, gender, and individual anatomy and are compromised by atherosclerotic occlusive disease. The common femoral artery bifurcates into the profunda femoral artery and the superficial femoral artery. The profunda is the second major contributor to the cruciate collateral system. The anatomic location of the origin of the profunda femoris artery is variable, and its ostium can be covered by a cannula placed too low in the common femoral artery. The remaining elements of the cruciate collateral system come from the medial and lateral circumflex branches of the common femoral artery, and flow to these collaterals can be disrupted by the cannula or in situ thrombosis after cannulation. [20] The superficial femoral artery descends to the popliteal artery, and this, in turn, leads to the tibial bifurcations where macro-emboli from the peri-cannula thrombotic material commonly lodge. The tibial arteries give rise to the dorsal pedal and medial and lateral tarsal vessels, which are considered end vessels and the site of lodgment of microthrombi. These microthrombi can come from the cannula from low flow distal to the cannula or from the systemic responses to ECMO.

Venous return from the limb occurs through the deep and superficial systems; within the deep system, venous blood exits the capillaries and follows a reverse track of the arteries, with the tibial veins merging to form the femoral veins and then ascending to the common femoral vein and the iliac venous system [20]. Additional drainage occurs through the superficial system, which joins the deep system at the popliteal fossa and the common femoral veins. The common femoral vein has a diameter range from 8 to 12 mm in adults. The External iliac vein is reported to have a diameter of 8 to 12 mm, similar to the common femoral veins, while the common iliac vein ranges from 10 to 15 mm in diameter [20]. These typical diameter measurements will be dependent on age, gender, and individual anatomy and are compromised by prior venous thrombotic events and prior indwelling cannulation leading to constrictive scar tissue.

In the leg, the muscle bundles reside in distinct compartments that can be bounded by fascia or by fascia and bone, which are sensitive to changes in intra-compartment pressures and lack the ability to expand significantly, which will induce increased compartmental pressure. The lower extremity consists of four compartments in the calf and three within the thigh [20]. The most vulnerable compartment is the anterior tibial compartment of the calf, which is bounded by the tibia, the fibula, the interosseous membrane, and the anterior fascia and is the commonest compartment to be involved in acute compartment syndrome. Increased pressure in a compartment of 30 mmHg for 6 h will lead to irreversible ischemia, rhabdomyolysis, nerve damage, and muscle necrosis. The development of necrosis will trigger a systemic inflammatory response syndrome (SIRS) or exacerbate a pre-existing SIRS [21].

## 3. Pathophysiology

**Figure 1:**
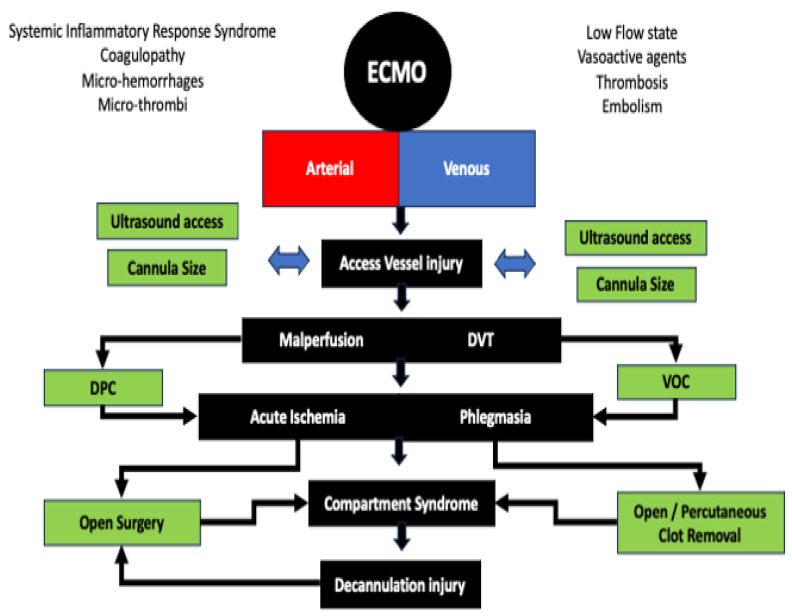
The etiology of arterial and venous complications induced by ECMO. With a background of systemic inflammatory response, arterial injury can be induced by anatomic injury, mechanical occlusion, thromboembolism with mal-perfusion, and subsequent compartment syndrome. Similarly, venous injury can lead to significant deep venous thrombosis and phlegmasia with subsequent arterial compromise and compartment syndrome. While many arterial and venous issues occur at cannulation, they can also occur during decannulation. DVT, Deep Venous Thrombosis; DPC, distal perfusion catheters; VOC, Venous Outflow Catheter.

The pathophysiology of vascular complications results from a series of interrelated events triggered by the implementation of ECMO (Figure 1). Arterial complications are isolated to VA-ECMO circuits, while venous complications are associated with both VA-ECMO and VV-ECMO.

### 3.1. Bio-Injury in ECMO

Circulation of blood through the membranes in an ECMO circuit and the shear stress of the flow in the system triggers a systemic inflammatory response, which induces the activation of the inflammatory, immunological, and coagulation cascades. Activation of the cascades promotes platelet activation, leukocyte recruitment, increased vascular permeability, coagulopathy, and endothelial dysfunction. These lead to microcirculatory dysfunction and induce aseptic parenchymal inflammation injury in multiple organs and multi-system end organ dysfunctions [22]. Within the leg, the end result of these contributes to macro- and micro-circulation endothelial activation and dysfunction, micro-hemorrhages, and micro-thromboses within the tissues of the leg [23,24,25,26]. The relationship between bio injury and vascular complications continues to evolve, and the extent of its contribution to leg complications has yet to be fully established.

### 3.2. Acute Arterial Thrombosis

Acute arterial thrombosis is characterized by the acute formation of a thrombus within an artery, leading to obstruction of antegrade blood flow [27]. The pathophysiology in ECMO that leads to acute arterial thrombosis involves multiple interrelated processes, including luminal obstruction from the cannula, endothelial injury with luminal thrombosis, ongoing activation of platelets, and coagulation cascades due to the systemic inflammatory response to ECMO, poorly developed collateral circulation, pre-existing athero-occlusive disease, unintentional obstruction of the profunda femoris artery and or ipsilateral internal iliac artery coupled to an ongoing low flow state and need for vasopressor support due to the primary indication for the ECMO run [14]. Additionally, pericatheter fibrin deposition and thrombosis increase with the duration of the catheter within the recipient artery.

### 3.3. Acute Arterial Embolism

Acute arterial embolism occurs distally in the leg during the placement of the cannula and from accumulated thrombosis surrounding the cannula during the course of the ECMO run [28]. The pathophysiology of acute arterial embolism involves the issues related to the thrombus formation around the cannula and the subsequent dislodgement of small or large fragments of the thrombus into the distal circulation of the leg, leading to distal tibial arterial occlusion and or occlusion of end vessels in the foot and calf muscles [28]. Embolism leads to tissue Ischemia and Infarction.

### 3.4. Compartment Syndrome

Acute ischemia in the leg can lead to the development of compartment syndrome, which is characterized by increased pressure (>30 mmHg) within closed anatomic spaces (compartments) in the leg, which leads to tissue ischemia and tissue infarction [29,30,31,32]. The sequelae of an ECMO run, bleeding, inflammation, or tissue swelling can develop within the compartments of the leg and can contribute to an increase in the volume of its contents. Overall, compartment syndrome represents a critical imbalance between compartment contents and the compartment’s capacity to accommodate them, leading to compromised tissue perfusion, ischemia, and potential tissue necrosis [33]. Sustained compartment syndrome leads to impaired blood flow, tissue Ischemia, neurological Impairment, and muscle infarction [33]. The presence of prolonged ischemia and compromised muscle in the leg further contributes to the ongoing systemic Inflammatory response induced by an ECMO run [21].

### 3.5. Acute Venous Thrombosis

Deep venous thrombosis (DVT) results from the formation of a thrombus within the deep veins of the legs or pelvis. The pathophysiology of acute venous thrombosis involves several interconnected processes classically known as Virchow’s Triad (Endothelial Injury, Stasis of Blood Flow, and Hypercoagulability), each of which occurs during an ECMO run [34,35]. The most significant sequala of lower extremity DVT is the development of two forms of phlegmasia—phlegmasia cerulea dolens (extensive DVT with compartment syndrome) and phlegmasia alba dolens (extensive DVT with acute arterial ischemia and an associated compartment syndrome) [36,37].

### 3.6. Acute Venous Thromboembolism

Venous Thromboembolism occurs when thrombotic material developed around the venous cannula or in its inflow veins is dislodged. Most commonly, the material enters into a drainage cannula, leading to pump thrombosis and oxygenator failure. A second destination for the material is the lungs, which results in acute pulmonary embolism [38].

### 3.7. Iatrogenic Injuries

Iatrogenic injuries usually occur during initial placement or at the time of removal of cannulas [39]. Reported injuries included focal or extensive dissection, vessel perforation or avulsion with immediate or delayed retroperitoneal bleeding/hematoma formation, pseudoaneurysm, and abrupt decannulation occlusion. Additionally, cannula misdirection has been reported [40]. Following decannulation, the development of pseudoaneurysm in the femoral artery has been reported, and decannulation from venous and arterial sites can result in groin hematomas [41,42]. Infection rates at the groin cannulation site have been reported from 7% to 20% [43]. Malnourishment and obesity increase the risk of groin infection.

## 4. Vascular Complications

The incidence of arterial events is more common than venous events during a run of ECMO [13]. Limb ischemia has been associated with worse outcomes, with related complications mainly due to insufficient limb perfusion or embolic events [15,44,45,46]. While on VA-ECMO, early vascular adverse events reported are acute limb ischemia, arterial thromboembolism, and compartment syndrome leading to potential limb loss; arterial injury at the time of cannulation is reported [47]. Finally, cannula site bleeding and hematoma are common occurrences [48]. Early complications of VV-ECMO are commonly in situ thrombosis and associated thromboembolism. Venous injury with or without perforation or branch avulsion and cannulation site bleeding have also been reported during VV-ECMO [9,10]. Late arterial complications reported include arterial bleeding, access site infection, access site fluid collections [9,10], thromboembolism, pseudoaneurysm, and arterial stenosis. Late venous complications include post-phlebitic syndrome, veno-thrombo-embolism (VTE), and access site complications [39].

### 4.1. Predisposing Factors

Multiple reports have identified several pre-existing conditions and periprocedural factors that contribute to the development of vascular complications. Pre-existing risk factors may indicate a higher risk of vascular complications in adults, female gender, younger age, the presence of diabetes, and the presence of atherosclerotic occlusive disease or veno-stenotic disease [14]. The traditional cardiovascular risk factors of hypertension, hyperlipidemia, atherosclerotic lower extremity occlusive disease, diabetes mellitus, smoking, and increased body mass index have not been shown to impact the prevalence of limb ischemia [14]. Additional contributing factors to vascular complications Include the use of inotropes, vasopressors, pre-ECMO coagulopathy, and the disease severity that necessitated ECMO [8]. A difficult bedside cannulation has been identified as a procedural risk factor. In general, the size of the cannula has been dictated by the need to obtain adequate flows within the circuit [49]. During the procedure, the size of cannulas and prolonged dwell time have been associated with an increased risk of acute limb ischemia [50]. The severity of Sequential Organ Failure Assessment (SOFA) scoring at ECMO cannulation has been shown to be directly associated with the risk of the development of acute limb ischemia [43].

### 4.2. Access

In general, ECMO requires an arterial inflow and venous outflow cannula to complete the circuit, and vascular access can be achieved at various locations in the body [51,52,53]. Arterial cannula sizing ranges from 15 to 21 French, and sizing of the venous cannula ranges from 19 to 29 French. In adults, the femoral vessels (common femoral artery and vein) are used initially due to ease of access [54]. Venous access is generally achieved percutaneously, whereas arterial access is obtained through an open surgical approach (i.e., cutdown) or percutaneously using the Seldinger technique with or without ultrasound guidance [54,55]. It is recommended that the ultrasound guidance should be used during percutaneous access so that the anatomy of the femoral vessels is fully visualized and, in particular, that the ostium of the profunda femoris artery is identified so that the initial needle access is placed so as not to obstruct the ostium of the profunda femoris artery [56] Fluoroscopic guidance of the cannula adds additional safety to the procedures.

### 4.3. Arterial Events

Regarding ECMO complications, Cheng et al. described that 10% of patients suffering acute limb ischemia will then develop lower extremity compartment syndrome, and up to 5% will progress to a lower extremity amputation [57]. There is significant variation in the application of distal perfusion arterial cannula (DPC) during VA ECMO, as well as in the cannula type and placement method(s) used [58]. Numerous analyses have shown that in comparison to no DPC usage in VA ECMO, a functioning DPC leads to an average of 16% reduction in acute limb ischemia incidence; this reduction does not impact overall mortality [17]. A vascular dead space can exist between the DPC and the arterial cannula within the femoral artery, which can lead to in situ thrombosis. Vascular complications are more frequent after decannulation of percutaneous access when compared to open surgical cutdown access (~15% vs. ~3%) [43], which could suggest that operative surgical cannulation may lead to a reduction in decannulation issues. Late arterial complications associated with ECMO include arterial pseudoaneurysms and stenoses in VA ECMO [17].

Complications at the access site constitute a critical source of comorbidity around catheter removal following VA-ECMO [59]. Percutaneous peripheral VA-ECMO cannulation has demonstrated a lower rate of access-site infection, similar ischemic and sensory-motor complication rates, and better 30-day survival compared to surgical cannula placement [43]. VA- and VV-ECMO decannulation can be achieved using either open or percutaneous approaches. Vascular complications at decannulation happen between 8% and 20% of the time [60]. Open surgical decannulation is performed in those patients who were cannulated via open technique or for failed attempted percutaneous decannulation closure. The combination of ultrasound vessel imaging and the “preclose” technique at the time of implantation can allow the successful subsequent removal of ECMO cannulas and management of the arterial or venous access sites without operative groin management [43,61]. There is a 10% failure for percutaneous closure techniques. In comparison to surgical repair, the “preclose” closure technique has demonstrated a similar vascular complication rate but a lower groin wound infection rate [62]. Reports have shown that surgical repair of arterial access sites has a relatively high subsequent surgical site infection (SSI) rate that is greater than that reported for VV-ECMO repair (~10%) [41,42]. For immunocompetent ECMO recipients, about half of these SSIs will arise late, greater than 30 days after decannulation. Typically, later groin complication issues (seromas, lymphoceles, late hematomas or infections, and other non-specified or mixed collections) are the most common complications reported. Surgical intervention is necessary in up to 25% of cases, with debridement and placement of a muscle flap being the most common corrective procedure. The development of groin complications arising subacutely and requiring surgical reintervention will lead to a longer length of stay (LOS) for the index admission [63,64].

### 4.4. Venous Events

Larger cannulas in the jugular or femoral veins can induce lower flow and may then drive thrombosis formation. Prolonged ECMO time has been demonstrated as a risk factor for deep vein thrombosis (DVT) in the vein(s) used for access, as has percutaneous technique, which is also a risk factor for increased bleeding occurrence from at sites [65]. One group has shown that ECMO patients suffer high rates of venous thrombosis of both the arms and legs (~60%) and overall VTE occurrence (~10%) [66]. A positive correlation exists between VTE incidence and ECMO duration. Another critical finding is that DVT after decannulation was typically treated with appropriate ongoing anticoagulation therapy [17].

## 5. Treatment Strategies

**Figure 2:**
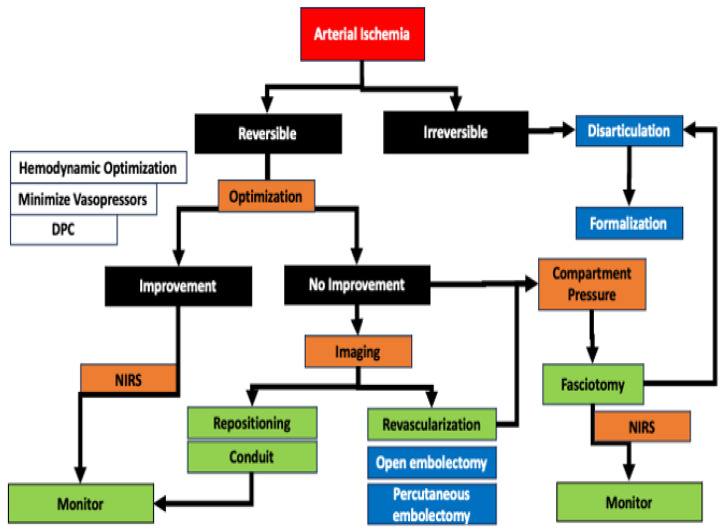
The management of arterial complications induced by ECMO. With a background of systemic inflammatory response, arterial injury can be induced by anatomic injury, mechanical occlusion, thromboembolism with mal-perfusion, and subsequent compartment syndrome. NIRS is a vital component for arterial surveillance on the arterial limb, and distal perfusion catheters (DPC) are an effective preventative strategy. If malperfusion is suspected in the presence of a DPC, the decision of whether the leg ischemia is reversible or irreversible is important early. Imaging, whether by duplex ultrasound or angiogram, can be performed in addition to systemic optimization of the patient to help with the diagnosis. Cannula repositioning and/or placement of a conduit is appropriate if the patient can tolerate a temporary cessation of ECMO. If imaging shows a thrombotic occlusion, open or percutaneous embolectomy is possible. In the presence of a compartment syndrome, fasciotomy of all compartments should be considered early. In the presence of irreversible ischemia, disarticulation at the ankle or knee is a rapid surgery with minimal blood loss and physiological stress that can temporize the patient, allowing for a planned but delayed formalization.

**Figure 3:**
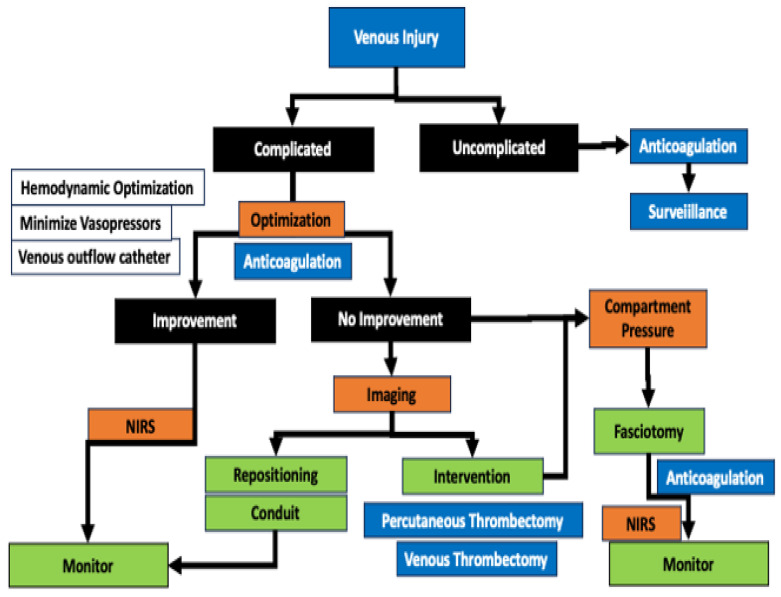
The management of venous complications induced by ECMO. Deep venous thrombosis is more likely due to systemic inflammatory response and low flow states. Venous injury is induced by the insertion of a catheter and the development of thrombosis due to mechanical thrombosis. Proximal thromboembolism can be performed from the tip of the catheter, while venous hypertension, phlegmasia, and subsequent compartment syndrome can occur when the tissue drains into the catheter site. The use of a venous outflow catheter can reduce venous hypertension. While many venous issues occur at cannulation, they can also occur during decannulation. Venous injury can be considered complicated and uncomplicated. The uncomplicated venous injury can be dealt with by systemic anticoagulation. The complicated venous injury will compromise tissue, and if anticoagulation and systemic optimization fail to lead to improvement in the limb, imaging by duplex or contrast venography should be performed. If imaging shows a thrombotic occlusion, open or percutaneous embolectomy is possible. In the presence of a compartment syndrome, fasciotomy of all compartments should be considered early. NIRS is a vital surveillance mechanism to ensure arterial perfusion is maintained in the presence of an extensive DVT.

Decisions around the treatment of a potentially threatened limb due to ischemia during a run of ECMO are based on the determination of a threatened salvageable limb from a non-salvageable limb (Figure 2 and Figure 3). The loss of previously detectable Doppler arterial signal suggests a threatened limb, while the absence of both arterial and venous Doppler signal indicated that the limb may be irreversibly damaged and non-salvageable [67]. The current vascular surgery literature defines a non-salvageable limb as one with absent arterial signals and only venous signals on Doppler ultrasound coupled to no motor or sensory function (Table 2). In contrast, a salvageable limb has arterial signs and has diminished but is not absent in sensory and motor function (Table 2). When compartment syndrome is suspected, the pressure in the compartment can easily be measured using a handheld manometer. The pressure of a tissue compartment normally falls between 0 and 8 mmHg. When the absolute pressure in the compartment is over 30 mmHg or the difference between the measured compartment pressure and diastolic blood pressure is <30 mmHg, compartment syndrome is present, and a fasciotomy is necessary [68].

### 5.1. Ischemia Secondary to Arterial Obstruction

In cases of ischemia due to obstruction from the cannula, placement of a DPC can ameliorate the situation if one has not been placed. In cases where a DPC is present, a limited angiogram of the leg should be performed to confirm the distal patency of the vessels. With patent vessels, moving the canulation site under controlled conditions to another vessel with a conduit is prudent.

### 5.2. Ischemia Secondary to Arterial Embolism

In cases of ischemia due to distal embolization, percutaneous or open intervention can be performed to remove the thrombus, and then the patient is maintained on the ECMO heparin regimen. If appropriate, an angiogram to confirm distal patency may be a valuable diagnostic adjunct to decide the limb’s salvageability. Sluggish flow in the calf suggests compartment syndrome. Lack of pedal vessel opacification is a predictor of poor limb salvage.

### 5.3. Ischemia Secondary to Arterial Thrombosis

Ischemia secondary to arterial thrombosis: In cases of ischemia due to a thrombus in the common femoral artery that limits perfusion, the placement of a DPC will improve the situation. In limbs that already have a DPC in place, a limited leg angiogram should be performed to confirm the patency of the vessels distally to the DPC. The DPC should be moved distally in the SFA to a patent vessel if necessary. At the time of decannulation, thrombo-endarterectomy with or without patch angioplasty will likely be required.

### 5.4. Venous Hypertension Due to Venous Obstruction

In cases of significant venous obstruction due to the presence of the venous cannula, a distal perfusion catheter can be inserted in the femoral vein and connected to the venous circuit to increase outflow from the leg. This uncommonly practiced intervention carries a risk of complications due to the need for negative access to the circuit.

### 5.5. Ischemia Due to Venous Thrombosis

In the rare case of phlegmasia without arterial ischemia, open or percutaneous thrombectomy can be performed and should be coupled with thigh and calf fasciotomies.

### 5.6. Compartment Syndrome

In patients where there is a concern for acute compartment syndrome in the calf or thigh, it may be challenging to ascertain clinical signs due to the patient’s condition, and thus, the use of a hand manometer is advised. If the reading suggests elevated compartment pressures, it is advisable to perform a four-compartment calf fasciotomy with or without a three-compartment thigh fasciotomy. These should be left open until the patients’ condition and extremity have normalized.

### 5.7. Major Amputation

Patients who develop irreversible ischemia are best served by a staged major amputation. Ankle disarticulation or knee disarticulation allows for a rapid, relatively bloodless amputation that is expeditious and can be achieved at the bedside if required. The wound can be managed easily until the patient hemodynamic and metabolic issues are resolved and suitable for a formalization as a below-knee or above-knee amputation.

## 6. Mitigation Strategies

### 6.1. Access Site Assessment

The patient’s history, physical exam, and imaging should be carefully reviewed to ensure the optimal limb is chosen for cannulation.

### 6.2. Ultrasound-Guided Access

The use of ultrasound guidance for percutaneous cannulation will aid the profunda femoris artery in the identification and preservation of its ostium without obstruction, thus maintaining a critical collateral flow pathway [69,70].

### 6.3. Cannula Size

To avoid a problem with large cannula placement, the body surface area (BSA) ratio to cannula size is greater than 11 [50]. If the calculated size of the cannula is inadequate for the required flows on the circuit, open surgical access with a conduit should be considered. Yang et al. noted that following surgical cutdown, there was a lower limb ischemia event rate (8.6%), which may, in part, be due to the surgical placement of the cannulas in association with an adjunctive DPC [16].

### 6.4. Distal Perfusion Catheter

Distal perfusion catheter (DPC) utilization has been shown to afford the necessary antegrade arterial perfusion and lower the limb ischemia rate; a 5- or 7 Fr antegrade DPC correctly located proximally in the superficial femoral artery (SFA) using ultrasound (US) optimized guidance and then perfused with arterial blood via a side port of the arterial cannula connecting to the DPC [71,72].

### 6.5. Thrombus Prevention

Systemic anticoagulation and a DPC (arterial) or a distal draining catheter (venous) can help prevent thrombus formation and enhance distal perfusion [73]. The ELSO anticoagulation guidelines recommend an initial bolus (50–100 IU/kg) at the time of cannulation, initiating an intravenous infusion of 5–20 IU/kg/h, and maintaining therapeutic anticoagulation goal at 20–50 IU/kg/h. Heparin administration has been the mainstay of anticoagulation in ECMO, but newer agents are now being reported [73,74]. The recommended anticoagulation goal ranges based on point-of-service activated clotting times (ACT) of 180 and 220 s. There is an increasing interest in the use of alternative and novel anticoagulants during ECMO [74,75,76]. In a recent systematic review (n = 307 patients), it was demonstrated that there were similar rates of complications (bleeding and thromboembolism) in patients who received either heparin or argatroban [77]. A second systematic review and meta-analysis (n = 847 patients) reported that the use of bivalirudin can significantly decrease major bleeding and thrombotic events and is a suitable alternative agent in the case of HIT and heparin resistance [76]. Currently, other direct thrombin inhibitors (dabigatran, desirudin, and lepirudin) have a limited or no role in anticoagulation during ECMO [74].

### 6.6. Improving Venous Drainage

The placement of a distal venous drainage catheter attached to the side port of the venous cannula has also been described as enhancing venous drainage and preventing venous hypertension and a low flow state that may precipitate thrombosis and venous ischemia. This intervention is not commonly employed due to its inherent risks [78].

### 6.7. Monitoring

NIRS monitoring combined with bedside Doppler interrogation allows early ischemia identification and initiation of measures to reverse ischemia. Baseline NIRS (tissue saturations StO2) of ≥40% and a distal perfusion pressure of at least 50 mmHg must be maintained to avoid limb ischemia [46,79]. NIRS monitoring of both instrumented and non-instrumented extremities for VA-ECMO recipients can differentiate between cannula-related obstruction (StO2 < 50% for over 240 s or StO2 offsets between the cannulated and non-cannulated legs > 15%) and other causes of hypoperfusion (a difference in StO2 between cannulated and non-cannulated leg < 15%) [79].

### 6.8. Re-Positioning or Revision of the Cannula Site

If there is increasing leg ischemia or an evident prolongation of the need for ECMO, revision of the arterial access site should be considered. The use of a T graft (i.e., a large conduit sewn to the femoral or axillary arteries so that a cannula is not inside the vessel lumen) may be considered. The creation of a subcutaneous T-graft enables flow into the ECMO cannula without jeopardizing blood flow to the limb, prevents the graft from being exposed, reduces the risk of dislodgment, and decreases the risk of infection [80].

## 7. Outcomes

The major outcomes related to limb ischemia during ECMO are increased mortality, loss of function, and major amputation. In a systematic review by Jia et al., the pooled incidence of early vascular complications was 29.5% (limb ischemia—12.6%; thromboembolism—6.8%; compartment syndrome—4.2%; fasciotomy—3.3%; Amputation—0.6%) and late vascular complications (arterial stenosis—7.6%; thromboembolism—6.8%, and pseudoaneurysm—1.3%) [17]. The clinical importance of placement of a pre-emptive DPC cannot be underestimated. Marbach et al. demonstrated in their meta-analysis of 22 studies that limb ischemia was reduced in patients who had a small arterial cannula in the femoral artery (OR 0.40, 95% CI 0.24–0.65; *p* < 0.001) and/or received a prophylactic DPC (OR 0.31, 95% CI 0.21–0.47; *p* < 0.001) [81]. Neither maneuver aimed at reducing limb ischemia resulted in a change in overall mortality [81]. In the meta-analysis by Jia et al., the odds of developing limb ischemia were 1.93 when patients did not have a preemptive DPC [17]. This was supported by the earlier work of Juo et al. [58] in their meta-analysis, which reported that the use of a DPC saw at least a 15.7% absolute reduction in the occurrence of limb ischemia compared to the absence of a DPC. The advent of acute limb ischemia is associated with increased patient mortality and a decreased survivor’s quality of life after ECMO [82]. The survival-to-discharge rate among VV-ECMO patients with lower extremity ischemia is reported in the range of 75%, which contrasts with less than 50% in patients placed on VA-ECMO patients.

## 8. Conclusions

Vascular complications during an ECMO run remain a significant issue with significant morbidity. The development of acute arterial ischemia is a harbinger of poorer long-term outcomes. A stepwise approach to mitigate these complications can reduce the impact of lower limb ischemia and should be incorporated into the care pathway of ECMO patients.

## Figures and Tables

**Table 1:** Complications associated with leg ischemia.

Systemic	Arterial	Venous
Bio-injury	Acute Arterial thrombosis	Acute Venous Thrombosis
Bleeding	Peri Cannula fibrin sheath and in situ thrombosis	Peri Cannula fibrin sheath and in situ thrombosis
Infection	Acute Arterial embolism	Venous thrombo-embolism
Multiorgan dysfunction	Malperfusion of the limb	Venous hypertension
	Compartment Syndrome	Compartment Syndrome
	Iatrogenic Insertion Injury	Iatrogenic Insertion Injury
	Iatrogenic Decannulation injury	Iatrogenic Decannulation injury

**Table 2:** Clinical parameters to allow differentiation of Salvageable vs. Non Salvageable extremities.

ECMO Perspective		Salvageable	Salvageable	Potentially Salvageable	Non-Salvageable
SVS Stage		I	IIa	IIb	III
Prognosis		Limb viable, not immediately threatened	Limb marginally threatened, salvageable with prompt revascularization	Limb immediately threatened, salvageable with immediate revascularization	Limb irreversibly damaged, major tissue loss or permanent nerve damage; revascularization is not appropriate
Findings	Sensory Loss	None	Minimal (toes)	More than toes, pain at rest	Profound, anesthetic
	Muscle Weakness	None	None	Mild or moderate	Paralysis (rigor)
Doppler Signal	Waveform	BiPhasic /Monophasic	Monophasic	Non	Non
	Arterial	Audible	Often inaudible	Inaudible	Inaudible
	Venous	Audible	Audible	Audible	Inaudible
Duplex		Normal flow	Deceased Flow	Decreased to intermittent Flow	Absent Pflow
Angiography		Normal Opacification and Normal Flow	Normal Opacification but Slow Flow	Reduced to No Distal Opacification with Slow Flow	No Opacification and No flow

SVS, Society for Vascular Surgery.

## Data Availability

Not applicable.
